# Aspirin use and long-term rates of sepsis: A population-based cohort study

**DOI:** 10.1371/journal.pone.0194829

**Published:** 2018-04-18

**Authors:** Joann Hsu, John P. Donnelly, Ninad S. Chaudhary, Justin X. Moore, Monika M. Safford, Junghyun Kim, Henry E. Wang

**Affiliations:** 1 University of Alabama School of Medicine, Birmingham, Alabama, United States of America; 2 Department of Emergency Medicine, University of Alabama School of Medicine, Birmingham, Alabama, United States of America; 3 Division of Preventive Medicine, Department of Medicine, University of Alabama School of Medicine, Birmingham, Alabama, United States of America; 4 Department of Epidemiology, University of Alabama at Birmingham, Birmingham, Alabama, United States of America; 5 Department of Medicine, Weill Cornell Medical College, New York, NY, United States of America; 6 Department of Emergency Medicine, University of Texas Health Science Center at Houston, Houston, Texas, United States of America; Kaohsiung Medical University Hospital, TAIWAN

## Abstract

**Objective:**

Sepsis is the syndrome of life-threatening organ dysfunction resulting from dysregulated host response to infection. Aspirin, an anti-inflammatory agent, may play a role in attenuating the inflammatory response during infection. We evaluated the association between aspirin use and long-term rates of sepsis as well as sepsis outcomes.

**Methods:**

We analyzed data from 30,239 adults ≥45 years old in the Reasons for Geographic and Racial Differences in Stroke (REGARDS) cohort. The primary exposure was aspirin use, identified via patient interview. The primary outcome was sepsis hospitalization, defined as admission for infection with two or more systemic inflammatory response syndrome criteria. We fit Cox proportional hazards models assessing the association between aspirin use and rates of sepsis, adjusted for participant demographics, health behaviors, chronic medical conditions, medication adherence, and biomarkers. We used a propensity-matched analysis and a series of sensitivity analyses to assess the robustness of our results. We also examined risk of organ dysfunction and hospital mortality during hospitalization for sepsis.

**Results:**

Among 29,690 REGARDS participants with follow-up data available, 43% reported aspirin use (n = 12,869). Aspirin users had higher sepsis rates (hazard ratio 1.35; 95% CI: 1.22–1.49) but this association was attenuated following adjustment for potential confounders (adjusted HR 0.99; 95% CI: 0.88–1.12). We obtained similar results in propensity-matched and sensitivity analyses. Among sepsis hospitalizations, aspirin use was not associated with organ dysfunction or hospital death.

**Conclusions:**

In the REGARDS cohort, baseline aspirin use was not associated with long-term rates of sepsis.

## Introduction

Sepsis is the syndrome of life-threatening organ dysfunction resulting from dysregulated host response to infection [1]. In the United States (US), sepsis is responsible for over 665,000 hospital admissions, 850,000 Emergency Department visits and 215,000 deaths annually [2, 3]. Current recommendations for treating sepsis are primarily aimed at recognition and management [4, 5]. Studies of other acute conditions, such as myocardial infarction (MI) and stroke, have highlighted the importance of prevention as a potential strategy for reducing the public health burden of fatal diseases. However, there have been few organized efforts targeting sepsis prevention.

Aspirin is an irreversible inhibitor of the enzyme cyclooxygenase, a key component in the activation of inflammatory pathways through the production of prostaglandins [6]. Aspirin therapy has been widely used for treating and preventing vascular diseases such as myocardial infarction and stroke [6–11]. The use of aspirin as a preventive therapy for sepsis is plausible and feasible. Sepsis impairs the hemostatic function of platelets, potentially mitigating hypercoagulability found in acute sepsis [12–14]. A limited body of research suggests that prior antiplatelet use is associated with decreased risk of mortality from sepsis [15–19]. There is limited knowledge of the association between baseline aspirin use and incidence of sepsis, and prior studies have been unable to link incident events with hospitalization outcomes [20, 21].

Similar to the effects seen in diseases such as MI and stroke, regular aspirin use could offer a potential sepsis prevention strategy. A better understanding of the association between regular aspirin use and long-term sepsis risk could set the stage for its use to prevent acute sepsis.

The Reasons for Geographic and Racial Differences in Stroke (REGARDS) is one of the nation’s largest population-based cohort studies, providing longitudinal data from community-dwelling individuals over a ten-year period. Using REGARDS data, we sought to determine if aspirin use is associated with long term rates of sepsis.

## Methods

### Study design

We performed a prospective cohort study using data from the Reasons for Geographic and Racial Differences in Stroke (REGARDS) cohort [22]. This study was approved by the Institutional Review Board of the University of Alabama at Birmingham.

### The REGARDS cohort

The REGARDS cohort is one of the nation’s largest population-based cohorts of community-dwelling adults. The parent study was designed to identify factors associated with racial and regional differences in stroke mortality. Between January 2003 and October 2007, 30,239 individuals aged 45 years or older were recruited. Participants were oversampled from the Southeastern US, with 30% of the participants recruited from the “Stroke Belt” (defined as the coastal plains of North Carolina, South Carolina, and Georgia), and 20% from the “Stroke Buckle” (the remainder of North Carolina, South Carolina, and Georgia, plus Tennessee, Mississippi, Alabama, Louisiana, and Arkansas). The cohort is 45% male and 42% African American and does not include Hispanics.

REGARDS collected baseline information on participant through telephone interviews and in-person assessments. Baseline information collected on each participant included medical and personal histories, demographic data, health behaviors, physical measures and vital signs, and a medication inventory. Blood and urine specimens were also collected from participants. Every six months, trained interviewers telephoned each participant to obtain updated information on all hospitalizations since the last follow-up call.

### Identification of aspirin use

REGARDS study personnel ascertained aspirin use during baseline subject enrollment in the study. We defined aspirin use based upon responses to the question, “Are you currently taking aspirin or aspirin containing products regularly, that is, at least two times each week?” REGARDS did not ascertain the reasons for aspirin use.

### Identification of sepsis events

The primary endpoint was hospitalization for sepsis. We identified all hospitalizations and Emergency Department visits attributed to a serious infection. To verify the presence of serious infection at admission and infection as a primary reason for hospitalization, two abstractors independently reviewed all relevant medical records. Abstractor agreement and additional physician-level review resolved any discordances. Sepsis was defined as infection at hospital presentation plus two or more systemic inflammatory response syndrome (SIRS) criteria, which include 1) heart rate >90 beats/minute, 2) fever (temperature >38.3°C or <36°C), 3) tachypnea (>20 breaths/min) or PCO_2_<32 mmHg, and 4) leukocytosis (white blood cells [WBC] >12,000 or <4,000 cells/mm^3^ or >10% band forms).

We assessed vital signs and laboratory findings within the first 28-hours of hospitalization to include Emergency Department care and up to one full day of inpatient care. Because the analysis focused on community-acquired sepsis, so we did not assess vital signs, laboratory findings, or development of sepsis at later time points. Initial review of 1,329 hospital records indicated excellent inter-rater consensus for the presence of serious infection (kappa = 0.92) and the presence of sepsis (kappa = 0.90) at the time of hospital presentation.

We did not include organ dysfunction in the primary definition of sepsis. In secondary analyses, we did evaluate death occurring during sepsis hospitalization.

### Covariates

We examined demographics, health behaviors, chronic medical conditions, Morisky medication adherence, and biomarkers as possible confounding variables in the relationship between aspirin use and sepsis. Demographic characteristics included age, sex, race, geographic region, annual household income, and highest level of education achieved. Health behaviors included smoking status and alcohol use. Chronic medical conditions included history of MI, coronary artery disease, atrial fibrillation, hypertension, stroke, chronic kidney disease, peripheral artery disease, chronic lung disease, deep vein thrombosis, dyslipidemia, diabetes, and obesity (S1 Table). Biomarkers considered potential risk factors for sepsis included hsCRP and ACR [23, 24]. REGARDS used a four-question version of the Morisky Medical Adherence Scale to determine participant’s medication compliance [25] (S2 Table). We defined medication adherence as 0 = good, 1 = fair, and 2–4 = poor.

### Data analysis

We used Pearson chi-square tests of association and t-tests of equal means to evaluate differences in participant characteristics between baseline aspirin users and non-users.

In order to assess the association between aspirin use and sepsis rates, we fit a set of multivariable Cox proportional hazards models, adjusting for demographics (age, sex, income race, geographical region, education), health behaviors (tobacco and alcohol use), chronic medical conditions (diabetes, hypertension, dyslipidemia, stroke, coronary artery disease, atrial fibrillation, deep vein thrombosis, peripheral artery disease, chronic kidney disease, chronic lung disease, obesity), Morisky Medication Adherence, and biomarkers (ACR, hsCRP). We verified the proportional hazards assumption using Schoenfeld residuals and tests of the interaction with the logarithm of follow-up time.

To evaluate the robustness of our results, we conducted a series of sensitivity analyses. We first repeated the analysis limited to sepsis events due lung, kidney and abdominal infections (the most common infection types). Because aspirin use may vary with risk of heart disease, we repeated the analysis stratified by a) history coronary artery disease (CAD) and b) Framingham Coronary Heart Disease (CHD) Risk Scores <10%, 10–20%, and >20%) [26].

Because aspirin use might be more likely among individuals with high comorbid illness burden, we defined a propensity score for baseline aspirin use for each participant using a logistic regression model and including the maximum number of covariates that would give a balance of factors between users and non-users [8–11, 27–29]. Specifically, the model included age, sex, race, region, education, smoking status, alcohol use, chronic kidney disease, chronic lung disease, deep vein thrombosis, dyslipidemia, hypertension, obesity, peripheral artery disease, CRP, Morisky Adherence Scale, and ACR [27]. We matched aspirin users and non-users 1:1 based upon propensity score, using nearest neighbor matching with a caliper equal to 30% of the standard deviation of the logit [30]. All analyses of the propensity-matched cohort were stratified by pair, to account for non-independence introduced by matching. We also assessed the association between aspirin use and sepsis incidence stratified by propensity score tertile [27, 31].

We repeated the analysis stratified by Morisky medication adherence classes. Finally, we performed a competing risks analysis, modeling all cause death as a competing event. Among first-sepsis hospitalizations, we also evaluated the associations between aspirin use and sepsis hospital death. In order to prevent over-fitting of the multivariable models, we included only hospital variables that were statistically significant on univariate analysis. We performed all analyses using Stata version 14.1 (Stata, Inc., College Station, Texas).

## Results

Out of 30,239 REGARDS participants, we included 29,690 with follow-up information available. Among these participants, 12,869 (43%) reported baseline aspirin use (Table 1). The median follow-up time was 6.2 years (IQR 5.1–8.1 years). Aspirin users were older at baseline and more likely to be male and white compared to non-users. Aspirin users were also more likely to have chronic medical conditions and to report good medication adherence.

**Table 1:** Characteristics of REGARDS participants, stratified by aspirin use.

Characteristic	AspirinUser (n = 12,869)	AspirinNon-User (n = 16,821)	p-value
**DEMOGRAPHICS**			
**Age** (mean ± SD)	66.7 ± 8.9	63.5±9.6	<0.001
**Sex**			<0.001
Male	6,715 (52.2)	6,619 (39.4)	
Female	6,154 (47.8)	10,202 (60.7)	
**Race**			<0.001
White	8,171 (63.5)	9,302 (55.3)	
Black	4,698 (36.5)	7,519 (44.7)	
**Income**			0.189
<$20,000	2,313 (18.0)	3,030 (18.0)	
$20,000–34,000	3,165 (24.6)	4,008 (23.8)	
$35,000–74,000	3,778 (29.4)	5,031 (29.9)	
≥ $75,000	2,071 (16.1)	2,626 (15.6)	
Unknown	1,542 (12.0)	2,126 (12.6)	
**Education**			<0.001
Less than high school	1,678 (13.1)	2,031 (12.1)	
High school graduate	3,288 (25.6)	4,378 (26.1)	
Some college	3,290 (25.6)	4,660 (27.7)	
College or Higher	4,602 (35.8)	5,740 (34.2)	
Missing	11 (0.1)	12 (0.1)	
**Region**			<0.001
Belt	4,421 (34.4)	5,864 (34.9)	
Buckle	2,831 (22.0)	3,384 (20.1)	
Nonbelt	5,617 (43.7)	7,573 (45.0)	
**HEALTH BEHAVIORS**			
**Tobacco Use**			<0.001
Current	1,705 (13.3)	2,580 (15.4)	
Past	5,723 (44.6)	6,197 (37.0)	
Never	5,402 (43.1)	7,979 (47.7)	
Missing	39 (0.3)	75 (0.5)	
**Alcohol Use**			<0.001
Heavy	513 (4.1)	664 (4.0)	
Moderate	4,427 (35.1)	5,265 (31.9)	
None	7,677 (60.9)	10,564 (64.1)	
Missing	252 (2.0)	328 (2.0)	
**Chronic Medical Conditions**			
Coronary Artery Disease	3,649 (28.9)	1,582 (9.6)	<0.001
Atrial Fibrillation	1,274 (10.2)	1,275 (7.8)	<0.001
Diabetes	3,652 (28.5)	3,052 (18.2)	<0.001
Hypertension	8,687 (67.7)	8,859 (52.8)	<0.001
Chronic Kidney Disease	1,765 (13.7)	1,484 (8.8)	<0.001
Peripheral Artery Disease	408 (3.2)	256 (1.5)	<0.001
Stroke	1,149 (9.0)	757 (4.5)	<0.001
Obesity	7,066 (55.0)	8,798 (52.4)	<0.001
Chronic Lung Disease	1,296 (10.1)	1,435 (9.5)	<0.001
Deep Vein Thrombosis	757 (5.9)	798 (4.8)	<0.001
Dyslipidemia	8,466 (67.8)	8,495 (52.8)	<0.001
**BIOMARKERS**			
**High Sensitivity C-Reactive Protein (hsCRP)**			<0.001
hsCRP >3.0	4,666 (38.6)	6,620 (42.1)	
Missing	793 (6.2)	1,105 (6.6)	
**Albumin-to-Creatinine Ratio (ACR)**			<0.001
ACR≥ 30	2,063 (16.8)	2,244 (14.0)	
Missing	600 (4.7)	806 (4.8)	
**Morisky Medical Adherence Scale**			<0.001
0 (good)	8,640 (70.6)	10,313 (70.0)	
1 (fair)	2,807 (22.9)	3,167 (21.5)	
2–4 (poor)	795 (6.5)	1,248 (8.5)	
Missing	627 (4.9)	2,093 (12.4)	

There were a total of 1,526 first sepsis events in this population. Of these hospitalizations, 1,122 (73.5%) presented with severe sepsis and 140 (9.2%) resulted in hospital death. The most common infection types associated with sepsis were lung, kidney and abdominal infections. (Table 2)

**Table 2:** Infection types of first-sepsis hospitalizations, stratified by aspirin use. Total of 1,526 first sepsis cases.

Infection Type	n (%)
Pneumonia	601 (39.4)
Urinary Tract Infections	260 (17.0)
Abdominal	231 (15.1)
Bronchitis	137 (9.0)
Skin	122 (8.0)
Sepsis	103 (6.8)
Fever of unknown origin	29 (1.9)
Catheter	6 (0.4)
Surgical	10 (0.7)
Meningitis	5 (0.3)
Other/Unknown	22 (1.4)

**Fig 1:**
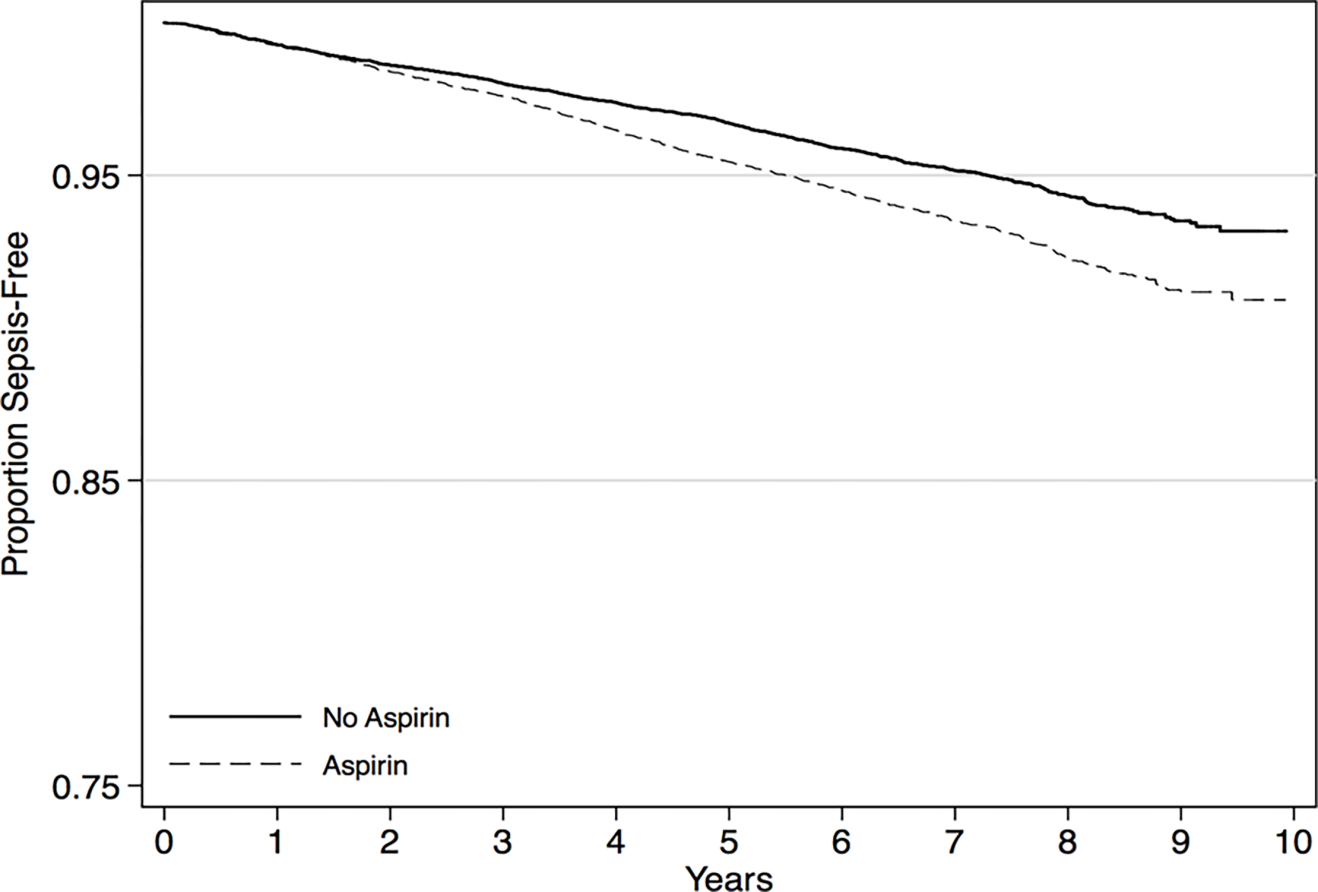
Kaplan Meier curve for first sepsis events, stratified by baseline aspirin use.

In unadjusted analyses, aspirin use was associated with increased rates of sepsis (Table 3: HR 1.35; 95% CI: 1.22–1.49) (Fig 1). However, after multivariable adjustment for potential confounding factors, the association between aspirin use and sepsis was attenuated (adjusted HR 1.00; 95% CI: 0.90–1.13).

**Table 3:** Multivariable Cox regression models evaluating associations between aspirin use and first sepsis events. ASA = aspirin.

Exposure	Total N	Event N (%)	Hazard Ratio (HR) (95% CI)			
			Crude	Model 1	Model 2	Model 3
**Baseline ASA**						
No ASA	16,821	748 (4.5)	Ref	Ref	Ref	Ref
ASA	12,869	778 (6.1)	1.35 (1.22–1.49)	1.18 (1.07–1.31)	1.19 (1.07–1.32)	1.00 (0.90–1.13)
**Prior Coronary Artery Disease (CAD)**						
No ASA	1,582	115 (7.3)	Ref	Ref	Ref	Ref
ASA	3,649	308 (8.4)	1.08 (0.87–1.34)	1.05 (0.85–1.31)	1.08 (0.87–1.35)	1.09 (0.85–1.40)
**Framingham Coronary Heart Disease (CHD) Risk Score**						
** <10%**						
No ASA	9,745	330 (3.4)	Ref	Ref	Ref	Ref
ASA	5,325	219 (4.1)	1.19 (1.01–1.42)	1.16 (0.98–1.38)	1.17(0.99–1.39)	1.10 (0.91–1.33)
** 10–20%**						
No ASA	3,022	173 (5.7)	Ref	Ref	Ref	Ref
ASA	2,218	119 (5.4)	0.90 (0.71–1.14)	0.94 (0.74–1.18)	0.97 (0.76–1.22)	0.89 (0.69–1.15)
** >20%**						
No ASA	1,570	98 (6.2)	Ref	Ref	Ref	Ref
ASA	1,191	100 (8.4)	1.31 (0.99–1.73)	1.29 (0.98–1.71)	1.27 (0.96–1.69)	1.14 (0.84–1.57)

Model 1 = Adjusted for demographics (age, sex, race, region, income, education)

Model 2 = Model 1 + Health Behaviors (alcohol use, smoking status)

Model 3 = Model 2 + Chronic Medical Conditions (diabetes, hypertension, dyslipidemia, stroke, coronary artery disease, atrial fibrillation, deep vein thrombosis, peripheral artery disease, chronic kidney disease, chronic lung disease, obesity), hsCRP, ACR, Morisky Adherence Scale

Models stratified by Framingham CHD Risk Score for these associations are

Model 1* = Adjusted for demographics (race, region, income, education)

Model 2* **=** Model 1* + Health Behaviors (alcohol use)

Model 3* = Model 2* + Chronic Medical Conditions (stroke, coronary artery disease, atrial fibrillation, deep vein thrombosis, peripheral artery disease, chronic kidney disease, chronic lung disease, obesity), hsCRP, ACR, Morisky Adherence Scale

Sensitivity analyses verified the primary results. When repeating the analysis limited to sepsis due to lung, kidney and abdominal infections, we observed similar results (adjusted HR 1.01; 95% CI: 0.88–1.15). On stratification by Framingham CHD Risk Score, we found that the association between aspirin use and sepsis were similar between CHD risk groups. (Table 3)

In the propensity-matched analysis, there were a total of 8,923 aspirin users matched 1:1 with non-aspirin users. In the matched cohort, there was no association between aspirin use and rates of sepsis (HR 0.97; 95% CI: 0.85–1.11). We also found no association between aspirin use and sepsis after stratifying by propensity score and Morisky medication adherence. (Table 4) When considering all cause death as a competing risk to sepsis hospitalization, the association between aspirin and sepsis was not statistically significant. Among first sepsis hospitalizations, aspirin use was not associated with sepsis or sepsis hospital death (adjusted HR 0.96; 95% CI: 0.66–1.40).

**Table 4:** Sensitivity analyses. Associations with first sepsis events.

Model	Crude HR (95% CI)	Adjusted HR (95% CI)
**Full Cohort (n = 29,690)**	1.35 (1.22–1.49)	1.00 (0.90–1.13)
**Stratified by Propensity Scores**^**1**^		
Low Propensity for ASA use	1.25 (0.96–1.60)	1.03 (0.79–1.35)
Medium Propensity for ASA use	0.99 (0.82–1.21)	0.91 (0.74–1.06)
High Propensity for ASA use	1.10 (0.93–1.31)	1.04 (0.87–1.25)
**Stratified by Morisky Adherence Scale**^**2**^		
Good Medication Adherence	1.02 (0.88–1.19)	1.01(0.86–1.18)
Fair Medication Adherence	1.13 (0.86–1.48)	1.14 (0.86–1.53)
Poor Medication Adherence	1.08 (0.70–1.68)	0.95 (0.60–1.51)
**Propensity-Matched Cohort (n = 17,846)**^**3**^	0.97 (0.85–1.11)	-
**Competing Risks Analysis** **(all-cause death as competing risk)**	1.33 (1.20–1.47)^4^	0.99 (0.88–1.12)^4^

^1^Model adjusted for demographics (age, sex, race, region, income, education), Health Behaviors (alcohol use, smoking status), Chronic Medical Conditions (diabetes, hypertension, dyslipidemia, stroke, coronary artery disease, atrial fibrillation, deep vein thrombosis, peripheral artery disease, chronic kidney disease, chronic lung disease, obesity), hsCRP, ACR, Morisky Adherence Scale

^2^Model adjusted for all of the above except Morisky Adherence Scale

^3^Propensity-matched model adjusted for age, sex, race, region, education, smoking status, alcohol use, chronic kidney disease, chronic lung disease, deep vein thrombosis, dyslipidemia, hypertension, obesity, peripheral artery disease, Morisky Adherence Scale, hsCRP, ACR.

^4^ Subdistribution Hazard ratio.

## Discussion

Using data from the REGARDS study, a large population-based cohort of community-dwelling adults in the US, we found no association between aspirin use at baseline and long-term rates of sepsis after adjustment for demographics, health behaviors, chronic medical conditions, and biomarkers. Furthermore, among first sepsis events, we found no association between aspirin use prior to hospitalization and odds of sepsis hospital death.

Prior studies investigating the influence of aspirin upon sepsis susceptibility and outcomes have reached varying conclusions. Wiewel, et al. conducted a prospective cohort study using data from 972 ICU patients and found that prior antiplatelet therapy was not associated with severity of sepsis, rates of septic shock, or sepsis mortality [21]. Using a nested cohort study, Al Harbi, et al. found that aspirin use in ICU patients was associated with increased rates of severe sepsis and length of stay [32]. In contrast to these prior studies, our data were not limited by ICU admission and included information on a range of baseline participant characteristics. We were able to define regular aspirin exposure as well as identify sepsis events occurring over a 10-year span. We note that the greatest attenuation of the association between aspirin and first sepsis events occurred after adjusting for demographics and chronic medical conditions, suggesting that aspirin use may be acting as a surrogate marker for comorbid burden.

Of note, we observed no association between baseline aspirin use and sepsis mortality, a finding that contrasts with several prior studies. However, these prior works had important methodological differences [16–19]. Falcone, et al. found that regular aspirin therapy was associated with decreased mortality due to community-acquired pneumonia [33]. However, pneumonia cases reflect only a subset of sepsis cases, and these results may not be generalizable to all sepsis outcomes. In a population-based cohort study in Taiwan, Tsai, et al. found that prior antiplatelet use was associated with lower rates of sepsis mortality [15]. However, the study only included antiplatelet use in the 30 days prior to admission, and did not differentiate aspirin from other antiplatelet agents. Similarly, in a meta-analysis, Trauer, et al. observed 7% lower sepsis mortality among pre-existing aspirin users, but most of the included studies did not differentiate between the use of aspirin and other antiplatelet agents [34]. Of note, in our series we observed a sepsis hospital mortality rate of only 9.2%, while the studies in the Trauer, et al. study reported sepsis mortality ranging from 14 to 42%. Thus, the observed associations may be due to the higher acuity sepsis populations included in the study.

Aspirin has multiple effects in critically ill patients as antiplatelet drug to inhibit platelet function to depress interaction of inflammatory mediators with immune cells therefore modulates the adverse effects associated with the inflammatory reactions [35–37]. In addition, aspirin stimulates the synthesis of nitric oxide that inhibits the interactions between cells, and decreases poly-morph neutrophil recruitment [38]. The lack of beneficial effect of aspirin may be a reflection of the interaction on these multiple pathways.

For the past decade, research and management efforts have been focused on the acute care of sepsis and infection. However, prevention can be a very powerful management strategy. Societal rates of MI, stroke, or cardiovascular disease mortality have been mitigated by organized prevention efforts [39]. In prior studies we have identified a host of novel risk factors associated with sepsis susceptibility [40]. Additional study must identify other potential interventions to limit and reduce long-term rates of sepsis.

## Limitations

We assumed that aspirin use was continuous throughout the 10-year follow-up period; true aspirin use may have fluctuated over time. In order to address the effects of variations in aspirin use, we controlled for medication adherence in our analyses. We used various methods to adjust for possible confounding by indication, but there could have been additional factors associated with aspirin use omitted from the analysis. We also did not have information on the indications for aspirin use. We surmised that aspirin use was for prevention of coronary heart disease, but participants may have taken aspirin for stroke prophylaxis or analgesia.

REGARDS includes only African American and White participants. We were able to obtain data on the presence or absence of chronic medical conditions, but not disease severity. The data did not include any information on immunocompromised participants. As the focus of our study was on community-acquired sepsis, we did not include cases of sepsis that occurred during hospital stay. Some participants were not admitted to the hospital and were treated only in the Emergency Department, but we reviewed their medical records to confirm that these events fulfilled sepsis criteria. We may not have been able to detect all sepsis events as REGARDS is not a surveillance study. We observed a sepsis death rate (9%) that is lower than in other studies.

## Conclusion

Aspirin use was not associated with long-term rates of sepsis in the REGARDS cohort.

## Supporting information

S1 Table Detailed definitions and technical information for sociodemographics, health behaviors, chronic medical conditions and biomarkers. (DOCX)

S2 Table Morisky medication adherence scale. (DOCX)
