# Supplementary material for: Aspirin use and long-term rates of sepsis: A population-based cohort study
Source: PLoS One. 2018 Apr 18;13(4):e0194829. doi: 10.1371/journal.pone.0194829 (PMC5905958; doi:10.1371/journal.pone.0194829)
Supplement: S1 Table — (DOCX) [file pone.0194829.s001.docx]

**S1 Table**

Detailed definitions and technical information for sociodemographics, health behaviors, chronic medical conditions and biomarkers in the REGARDS Cohort.

| **Variable** | **Definition and/or Technical Information** |
| --- | --- |
| **Sociodemographics** |  |
| Age | Age in years, dichotomized to ≥75 years vs <75 years |
| Gender | Male, female |
| Race | African American, white |
| Education | Participant reported:   - Less than high school - High school graduate - Some college - College or higher - Missing |
| Income | Participant reported:   - <$20k - $20k-$34k - $35k-$74k - ≥$75k - Missing (not reported) |
| Geographic Region | Participant residence:   - Stroke Buckle (coastal plains of North Carolina, South Carolina and Georgia) - Stroke Belt (remainder of North Carolina, South Carolina and Georgia, plus Tennessee, Mississippi, Alabama, Louisiana and Arkansas) - Non-Belt/Buckle (other states) |
|  |  |
| **Health Behaviors** |  |
| Smoking Status | Participant reported:   - Current - Past - Never |
| Alcohol use | Participant reported:   - None - Moderate (up to 1 drink per day for women or 2 drinks per day for men) - Heavy (>1 drink per day for women and >2 drinks per day for men). |
|  |  |
| **Chronic Medical Conditions** |  |
|  |  |
| Coronary Artery Disease | Participant reported history of myocardial infarction, coronary artery bypass grafting, or cardiac angioplasty or stenting, or baseline electrocardiographic evidence of myocardial infarction. |
| Atrial Fibrillation | Participant reported history of atrial fibrillation. |
| Hypertension | Systolic blood pressure ≥140 mm Hg, diastolic blood pressure ≥90 mm Hg, or participant reported antihypertensive agent use. |
| Stroke | Participant reported history of stroke or transient ischemic attack. |
| Chronic Kidney Disease | eGFR <60 mL/min/1.73m^2^.(1)  Assay by colorimetric reflectance spectrophotometry (Ortho Vitros Clinical Chemistry System 950IRC, Johnson & Johnson Clinical Diagnostics, Raritan, New Jersey, USA). eGFR based upon CKD-Epi equation. |
| Peripheral Artery Disease | Participant reported history of lower extremity arterial bypass or leg amputation. |
| Chronic Lung Disease | Participant use of pulmonary medications (beta agonists, leukotriene inhibitors, inhaled corticosteroids, combination inhalers, ipratropium, cromolyn, aminophylline and theophylline) as a surrogate for chronic lung disease. |
| Deep Vein Thrombosis | Participant reported history of deep vein thrombosis. |
| Dyslipidemia | Low-density lipoprotein cholesterol >130 mg/dL or participant reported use of lipid lowering medications. |
| Diabetes | Fasting glucose ≥126 mg/L (or a glucose ≥200 mg/L for those not fasting) or participant reported use of insulin or oral hypoglycemic agents. |
| Obesity | Waist circumference [>102 cm for males or >88 cm for females] or body mass index ≥30 kg/m^2^.(2) |
| **Biomarkers** |  |
| Albumin-to-Creatinine Ratio (ACR) | Abnormal defined as ACR ≥30 mcg/mg.  Albumin assay by nephelometry (BN ProSpec Nephelometer, Dade Behring, Siemens Healthcare, Deerfield, Illinois, USA). Urinary creatinine assay determined by rate blanked Jaffé procedure (Modular-P analyzer, Roche/Hitachi, Roche Diagnostics, Indianapolis, Indiana, USA). |
| High Sensitivity C-Reactive Protein (hsCRP) | Abnormal defined as hsCRP >3.0 mg/dL.(3)  Assay by particle-enhanced immunonephelometry (N High-sensitivity CRP, Siemens AG, Munich, Germany). |

**References**

1. Levey AS, Stevens LA, Schmid CH, Zhang YL, Castro AF, 3rd, Feldman HI, et al. A new equation to estimate glomerular filtration rate. Ann Intern Med. 2009;150(9):604-12.

2. Pampallona S, Tsiatis AA. Group sequential designs for one-sided and two-sided

hypothesis testing with provision for early stopping in favor of the null hypothesis. J Stat Planning and Inference. 1994;42:19-35.

3. Ridker PM, Rifai N, Clearfield M, Downs JR, Weis SE, Miles JS, et al. Measurement of C-reactive protein for the targeting of statin therapy in the primary prevention of acute coronary events. N Engl J Med. 2001;344(26):1959-65.
