# Supplementary material for: Aspirin use and long-term rates of sepsis: A population-based cohort study
Source: PLoS One. 2018 Apr 18;13(4):e0194829. doi: 10.1371/journal.pone.0194829 (PMC5905958; doi:10.1371/journal.pone.0194829)
Supplement: S2 Table — (DOCX) [file pone.0194829.s002.docx]

**S2 Table**

Elements of the 4-item Morisky Medication Adherence Scale: (25)

1. Do you ever forget to take your medicine?
2. Are you careless at times about taking your medicine?
3. When you feel better, do you sometimes stop taking your medicine?
4. Sometimes if your feel worse when you take the medicine, do you stop taking it?
